# The NLRP3 inflammasome as a new target in respiratory disorders treatment

**DOI:** 10.3389/fimmu.2022.1006654

**Published:** 2022-09-20

**Authors:** Katarzyna Leszczyńska, Dominika Jakubczyk, Sabina Górska

**Affiliations:** Laboratory of Microbiome Immunobiology, Hirszfeld Institute of Immunology and Experimental Therapy, Polish Academy of Sciences, Wrocław, Poland

**Keywords:** NLRP3 inflammasome, allergic rhinitis, allergic asthma, chronic obstructive pulmonary disease, probiotics, IL-1β, treatment, anti-inflammatory

## Abstract

In recent years a continuous increase in new cases of respiratory disorders, such as rhinitis, asthma, and chronic obstructive pulmonary disease (COPD), has been observed. The exact pathomechanism of these diseases is still blurry, resulting in the lack of targeted and effective therapy. The conventional use of treatment strategies, such as antihistamine drugs and/or glucocorticosteroids act mainly symptomatically and have significant side effects. Specific allergen immunotherapy is only useful in the management of specific allergies and selected patients. Therefore, new therapeutic solutions are constantly being sought. The novelty of recent years has been the association between NLRP3 inflammasome activation and the development of airway inflammatory diseases. This seems to be an interesting therapeutic target that may support or even replace traditional therapies in the future. The review presented, discusses the contribution of NLRP3 inflammasome to the development of allergic rhinitis, allergic asthma, and COPD. Moreover, the modulatory properties of probiotics as potential inhibitors of NLRP3 inflammasome are emphasised.

## 1 Introduction

Respiration is one of the basic physiological processes that enable organisms to survive. The airways are faced with millions of stimuli every day, which include allergens, bacteria, fungi, viruses or air pollution. The proper response for these agents is provided by the T cells, macrophages, dendritic cells and the airway epithelium (1). However, the airway’s homeostasis and integrity can be diminished, which leads to an inflammatory state. Acute inflammation might be considered as a beneficial process as it leads to the activation of the defence processes. It initiates wound healing, prevents penetration of foreign particles into the internal environment of the organism by phagocytic cells’ activation, and helps to maintain the homeostasis of the tissue (2, 3). Acute inflammation starts rapidly and persists for a few days depending on the trigger factor, whereas chronic inflammation is a slower process and long-lasting state. It leads to the influx of the inflammatory cells into the injured spot, enhancement of the inflammatory processes and finally tissue destruction (2, 4). The inflammatory cascade is powered by cytokines, especially IL-1β and IL-18, which are activated by the protein complex, called the inflammasome.

The inflammasomes were identified by Martinon and co-workers in 2002 as a ‘caspase-activating complex’ (5), starting a new chapter in the understanding of innate immunity and inflammatory diseases. Inflammasomes are multi-protein complexes, playing a crucial role in innate immunity and are involved in the regulation of inflammatory processes activated during infection or cellular damage. The many kinds of inflammasomes e.g. NLRC4, NLRP1, NLRP6, AIM2, and IFI16 have been identified (3), however, the most studied inflammasome is NLRP3 (NOD-, LRR- and pyrin domain-containing protein 3).

The review aims to describe and summarise the contribution of NLRP3 inflammasome to respiratory disorders development, including allergic rhinitis, asthma and chronic obstructive pulmonary disease (COPD). These diseases remain of scientific interest because of the continuously increasing incidence rate. However, the complex background impedes clearly identifying the causes and describing their exact pathomechanism. The studies highlighting the impact of inflammasome on chronic inflammatory disorders are the novelty of recent years. Therefore, the disease entities mentioned above were selected due to the considerable scientific interest in inflammasome contribution in their pathomechanism. We also discussed the potential therapeutic substances that can act as a specific antagonist of the NLRP3 inflammasome and diminish airway inflammation. Finally, we considered using probiotics to treat respiratory diseases *via* modulating the inflammasome signalling pathway.

## 2 Mechanism of the NLRP3 inflammasome’s activation

The NLRP3 inflammasome is a protein complex located in the cytosol, which consists of three interacting proteins:

1. NLRP3 (Cryopyrin, PYPAF1) which acts as a sensor, and contains the N-terminal pyrin domain (PYD), central NACHT domain (nucleotide-binding oligomerization domain) and C-terminal leucine-rich repeat domain (LRRs) (6);
2. ASC- is an adaptor part composed of PYD and CARD (cysteinyl aspartate-specific proteinase (caspase) recruitment domain) through which it interacts with NLRP3 and Caspase-1 respectively (6);
3. Procaspase-1 acts as an effector (7–9), it is synthesised as an inactive proenzyme, which is self-activated by proteolytic cleavage. The mature form of the enzyme is composed of 10- and 20-kDa subunits. The activated Caspase-1 processes the pro-IL-1β and pro-IL-18 into their active forms, which afterwards are secreted by cells (10).

**Figure 1:**
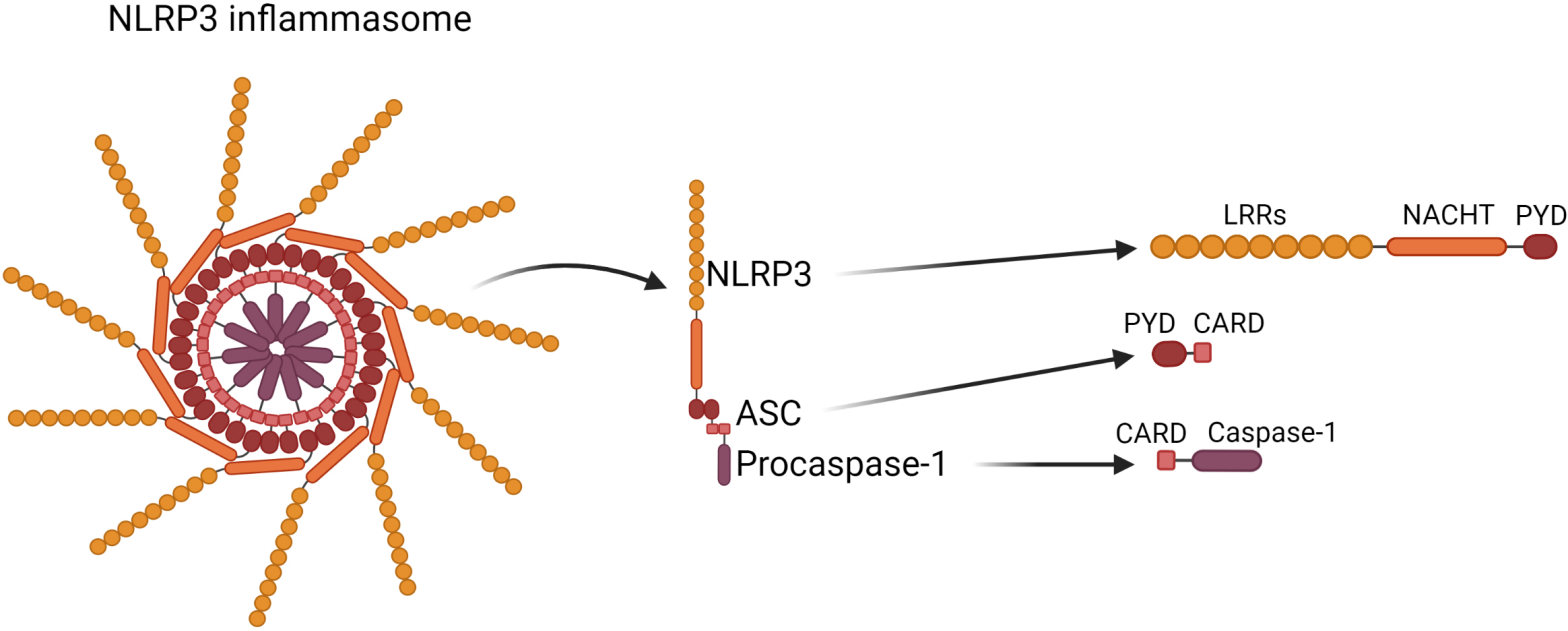
Structure of the NLRP3 inflammasome. NLRP3 inflammasome consists of NLRP3 contains the N-terminal pyrin domain (PYD), central NACHT domain (nucleotide-binding oligomerization domain) and C-terminal leucine-rich repeat domain (LRRs); ASC, which is composed of PYD and CARD (cysteinyl aspartate-specific proteinase (caspase) recruitment domain) through which it interacts with NLRP3 and Caspase-1 respectively; Caspase-1 which is composed of 10- and 20-kDa subunits.

The structure of the NLRP3 inflammasome was shown in **Figure 1**.

The NLRP3 inflammasome belongs to pattern recognition receptors (PRRs). The PRRs recognize pathogen-associated molecular patterns (PAMPs) and damage-associated molecular patterns (DAMPs), which trigger the defence’s mechanisms to eliminate infection and/or repair damaged tissue (11). NLRP3 is activated in response to various stimuli, such as pathogens or cell death. However, the NLRP3 inflammasome requires specific activation signals to achieve its functionality. The ‘two-signals’ model (known as a ‘canonical pathway’) for the NLRP3 inflammasome activation is commonly accepted. It consists of two stages. The priming signal occurs first, which leads to activation of the NF-κB transcription followed by upregulating of the NLRP3, pro-IL-1β and pro-IL-18 expression. The priming signals can be ligands for TLRs (toll-like receptors), NLRs, or pro-inflammatory cytokines such as TNF-α or IL-1β. Moreover, there are also transcription-independent signals which promote the NLRP3 activation, including deubiquitination *via* BRCC3 (the deubiquitinating enzyme) or phosphorylation by JNK-1 (11).

The second signal leads to the assembly of the protein complex and the final activation of NLRP3. There is a varieties galore of stimuli that can activate the NLRP3 inflammasome. These include bacterial and fungal components and toxins, pathogen-associated RNA, ATP, K^+^ ionophores, reactive oxygen species (ROS) or lysosomal damage (8, 11, 12). Active NLRP3 recruits Procaspase-1, and cleaves it to its active form. Caspase-1 processes pro-IL-1β and pro-IL-18 into their active form IL-1β and IL-18 respectively, which are secreted into the extracellular space, where they perform their further immune functions (8). Caspase-1 also processes the gasdermin D (GSDMD) and allows it to form pores within the plasma membrane, inducing a lytic, pro-inflammatory type of cell death known as pyroptosis. Pyroptosis enables the release of pro-inflammatory cytokines as well as intracellular pathogens, which then affect the spread of inflammation and prepare the immune system to respond to the infection (11).

**Figure 2:**
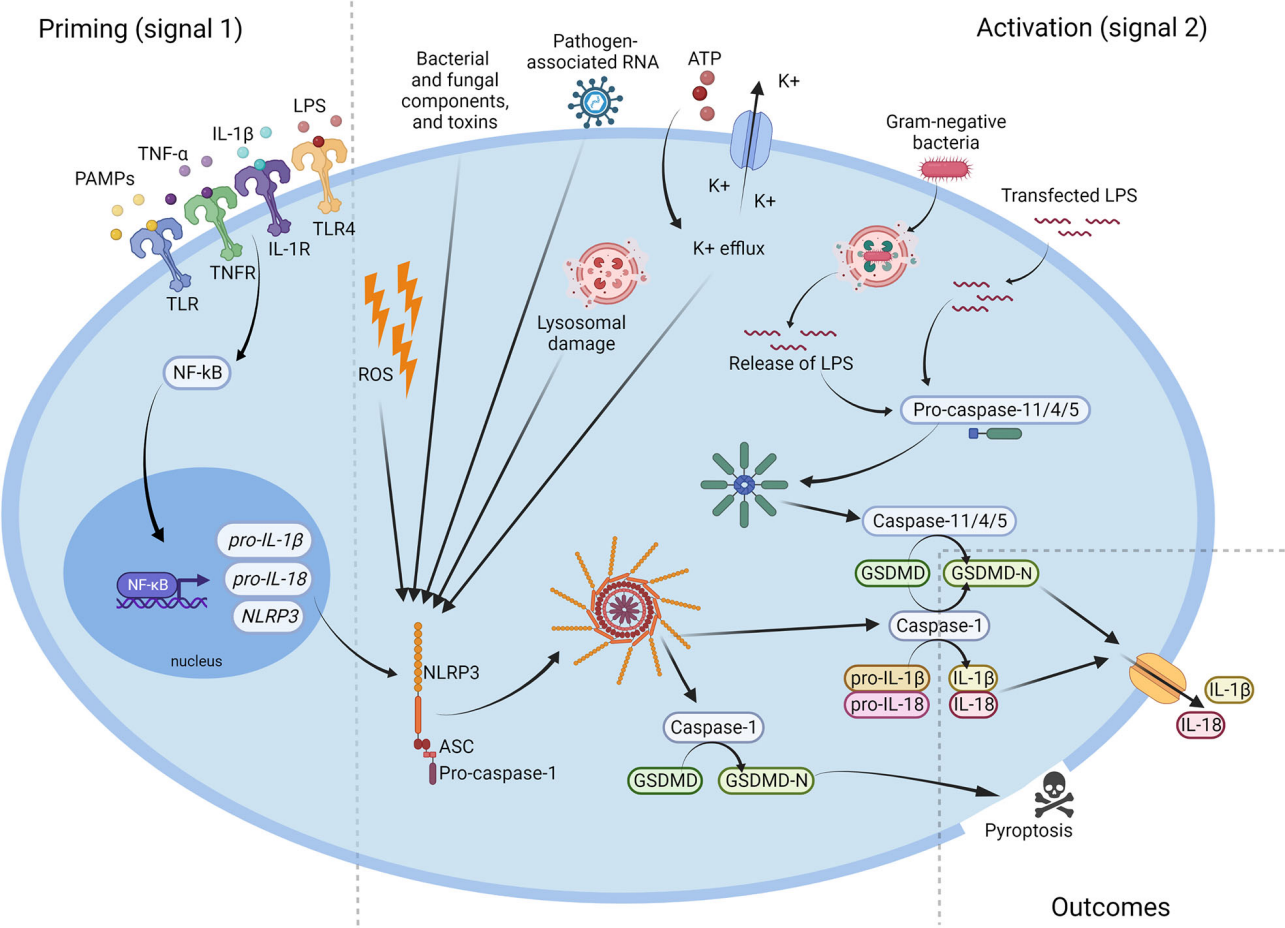
Activation of the NLRP3 inflammasome. Microbial components and endogenous cytokines provide the priming signal (signal 1) which activates the transcription factor NF-κB and leads to overexpression of NLRP3, pro-IL-1β, and pro-IL-18. Activation signal (signal 2) is provided by bacterial and fungal components and toxins, pathogen-associated RNA, ATP, K+ efflux, reactive oxygen species (ROS), or lysosomal damage, and leads to the activation of NLRP3 inflammasome and release of active Caspase-1. Caspase-1 cleaves the pro-IL-1β, pro-IL-18 and GSDMD to their mature forms, which are then secreted extracellularly. NLRP3, NOD-like receptor family pyrin domain containing 3; ATP, adenosine triphosphate; PAMP, pathogen-associated molecular pattern; ROS, reactive oxygen species; IL, interleukin; LPS, lipopolysaccharide; GSDMD, gasdermin D.

It has to be underlined that there is more than one mechanism of NLRP3 activation. In addition to the canonical activation pathway described above, there is also a ‘non-canonical’ NLRP3 inflammasome activation channel. Even though both, the canonical and non-canonical activation, lead to cell lysis and the release of proinflammatory cytokines, the mechanisms are significantly different (11, 12). The non-canonical NLRP3 inflammasome activation is induced by caspase-4/5 in humans and by Caspase-11 in mice. However, for mice, in which Caspase-11 is expressed at a low level in the cells, the activation of the NLRP3 inflammasome requires a priming signal to induce transcription of Caspase-11 as in the canonical activation pathway. In humans, caspase-4/5 is constitutively expressed, whereby the cytosolic LPS can activate the inflammasome without a priming signal (12). As the non-canonical inflammasome can recognize only gram-negative bacteria, LPS is the main activator of this path (11, 12). Intracellular LPS is recognized by the CARD domain of caspase-4/5 and/or Caspase-11, and this leads to oligomerization of those caspases. Further, caspase-4/5 and/or Caspase-11 cleave the gasdermin D, which targets the plasma membrane and forms pores. Pores in the cell membrane enable potassium efflux, pyroptosis and NLRP3 inflammasome activation. However, caspase-4/5 and Caspase-11 do not cleave interleukin-1β and interleukin-18. NLRP3 inflammasome activation by potassium efflux induces Caspase-1 production, IL-1β and IL-18 cleavage, and their secretion (11, 12). The mechanism of NLRP3 inflammasome’s activation was shown in **Figure 2**.

The NLRP3 inflammasome, as a protein complex, also undergoes post-transcription modifications, which regulate its activity. The most characterised are ubiquitination and phosphorylation (11). There are additional different factors, such as BRCC3, DUB enzyme, neurotransmitter dopamine (DA), cAMP, ubiquitin ligase TRIM31, FBXO3 (12), Bruton’s tyrosine kinase (13) or melatonin (14) regulating the NLRP3 inflammasome activity.

## 3 NLRP3 inflammasome in selected respiratory diseases

### 3.1 NLRP3 inflammasome in Allergic Rhinitis

#### 3.1.1 Allergic rhinitis – A disease overview

Rhinitis is a common term that describes upper airway disease, which is characterised by inflammation of nasal mucosa. The main symptoms include runny nose/rhinorrhoea, nasal itching, sneezing, nasal obstruction and others. Since rhinitis is a heterogeneous group of diseases (which include: 1) non-allergic and non-infectious rhinitis, 2) infectious rhinitis and 3) allergic rhinitis), a careful differential diagnosis is necessary (15–20).

Allergic rhinitis concerns around 10% - 30% of the adult population and around 40% of children in the United States (15). According to the Allergy & Asthma European Parliament Interest Group, even 100 million Europeans suffer from AR (16), and the socioeconomic burden has been increasing over the years (17, 18). The unequivocal causes of the increase in AR incidence are not entirely clear. However, it is assumed that the interaction between genetic composites and environmental factors plays a crucial role.

In general, AR is an inflammatory disease, mediated by IgE in hypersensitivity type I reaction. Briefly, the first contact with the allergen, in predisposed patients, leads to sensitisation. At this stage, the allergen comes into contact with antigen-presenting cells (APC) *via* a destroyed nasal epithelial barrier. The activated epithelial cells release, for instance, the IL-33, IL-25, and thymic stromal lymphopoietin (TSLP), leading to lymphoid activation and Th2 skewing (19). The APC (macrophages, dendritic cells) cells process the antigens and present them to the T cells. Recently, it has been shown that group 2 innate lymphoid cells (ILC2s), which are a rich source of cytokines (IL-4, IL-5, IL-9, IL-13) and can present antigens directly to the T cells are involved in the development of the Th2 immune response (20). The matured T cells release proinflammatory cytokines such as IL-4, IL-5, IL-9, and IL-13, which also stimulate B cells to produce the IgE specific antibodies (sIgE) instead of IgG (19). The sIgE antibodies are present in a free form in the blood, or they are anchored on the surface of mast cells within the mucosal surface. The allergic reaction is triggered each time the IgE antibodies recognize the allergen, causing the release of mediators such as histamine, leukotrienes, and prostaglandins from activated mast cells and basophils. This leads to the further infiltration of the eosinophils, neutrophils, basophils and Th2 cells into nasal mucosa, triggering an inflammatory state, and characteristic AR symptoms (21). Generally, AR can be classified as temporary or permanent. However, this is not a universal division as the same allergen may persist for different periods in different regions. Additionally, AR patients with reactions to many seasonal allergens may manifest symptoms throughout the year with various strengths. Therefore, to standardise and thus facilitate the diagnosis, the division of AR according to ARIA (The Allergic Rhinitis and its Impact on Asthma) was introduced (22). According to the guidelines, intermittent allergic rhinitis is manifested for no longer than 4 days per week or 4 consecutive weeks, whereas the persistent form manifests the symptoms longer than 4 days per week or 4 consecutive weeks. The symptoms are classified as mild (enable normal functioning) or moderate-severe (disrupt normal functioning) (22, 23). Due to the heterogeneity of the AR, it is important to identify the molecules-inducing symptoms, for example, using a skin prick test or determining the sIgE circulating in the blood in a free form. Nevertheless, in recent years, the phenotype called LAR (local allergic rhinitis) has been described (24, 25). The non-atopic patients with allergic rhinitis symptoms are positive for the allergen provocation test, Th2 skewing markers, and nasal secretion of sIgE, but negative for the skin prick test and serum sIgE, which are currently the basis of allergy diagnostics (25). This phenomenon underlines the complexity of the AR pathomechanism, the role of the epithelial barrier and mucus in the pathogenesis of AR, as well as indicates the need for estimation of a new diagnostic approach.

#### 3.1.2 Allergic rhinitis - Link with NLRP3 and IL-1β

AR is a complex issue and, despite much research into the pathomechanism of the disease, there are still many unknowns. The multitude of varieties of rhinitis makes it necessary to search for new markers of the disease and open up to new research trends. According to the available literature, IL-1β and inflammasome activation are crucial in the development of AR, therefore, they could be used for disease identification and as a therapeutic target respectively. Indeed, Han et al. indicated that the serum level of IL-1β could be a good marker of acute AR, as it was significantly increased in the group of children with moderate to severe persistent allergic rhinitis compared to children with intermittent allergic rhinitis (IAR) and mild persistent allergic rhinitis (26). Moreover, paternal allergic rhinitis and high expression of IL-1β were the risk factors for moderate to severe AR (6.4-fold and 4.7-fold increase in risk, respectively), and promoted the inflammation processes (26). Shi et al. (27) were interested in investigating the relationship between NLRP3 expression and severity of AR symptoms in patients. PBMCs (peripheral blood mononuclear cells) and monocytes/macrophages were isolated from patients with persistent moderate-severe AR and healthy control and were stimulated with LPS. They observed that mRNA expression levels of *NLRP3* and *IL-1β* and the level of produced IL-1β were significantly upregulated in the monocytes/macrophages and PBMCs of patients with AR compared with healthy controls (27). Interestingly, they also investigated the importance of mitochondrial ROS on IL-1β production. The LPS-primed PBMCs from AR patients and healthy control were pre-treated with ATP or ATP with Mito-TEMPO (mitochondrial ROS production inhibitor) indicating a higher generation of mitochondrial ROS in cells from patients with AR when compared to the healthy control. As they reported, the level of mitochondrial ROS was raised in LPS-primed PBMCs from AR patients and ATP-induced IL-1β secretion was inhibited in LPS-primed PBMCs in Mito-TEMPO groups. This suggested that the development of AR could be dependent on the ROS increase, which in turn, led to the activation of NLRP3 inflammasome and increased the level of IL-1β in nasal epithelial cells (27). Consistent results were obtained by Li et al. on human nasal mucosa samples (hNEC) (28) showing that an ozone-oxidised black carbon (BC) and pollen exposure led to oxidative stress by increasing the level of ROS production. Consequently, it increased the mRNA and protein levels of NLRP3 and IL-1β. However, pre-treatment with N-acetyl-L-cysteine (NAC – the ROS inhibitor) before BC and pollen stimulation reduced the relative level of ROS, mRNA expression and protein levels of NLRP3 and IL-1β. Additionally, pre-treatment with MCC950 (NLRP3 inhibitor) or YVAD (Caspase-1 inhibitor) did not alter the BC and pollen-induced synthesis of ROS. However, the level of IL-1β (mRNA and protein) was decreased, indicating that IL-1β secretion was dependent on NLRP3 inflammasome assembly induced by BC and pollen exposure. These results revealed that ROS production could activate the NLRP3 inflammasome, which led to an increasing level of IL-1β in the human nasal mucosa (28).

As already mentioned in paragraph 2, it is assumed that the activation of the inflammasome is based on a canonical (LPS, cell damage) or a non-canonical (caspase-dependent) pathway. However, it has recently been shown that IL-17, besides being an important factor in AR development (27, 29, 30), seems to impact the NLRP3 activation as well. The results obtained by Shi et al. showed that IL-17A may be involved in the pathogenesis of AR as its level was increased in AR patients and, interestingly, positively correlated with an IL-1β level (27). Quan et al. (29) investigated the importance of IL-17A in the OVA-induced AR mice model (IL-17A-deficient mice (KO) and WT mice) and reported that the level of IL-17A as well as IL-1β, and IL-4, IL-5, TNF-α decreased in KO mice compared with WT group. It was suggested that IL-17A plays a significant role in the development of AR by increasing proinflammatory cytokines (TNF-α and IL-1β) and skewing immune response into the Th2 (29). Both IL-1β and IL-17, seem to be involved in the pathogenesis of allergic rhinitis, and these two signalling pathways can be associated (27).

Another aspect that could be raised in the context of NLRP3 activation is the relationship between IL-1β and IL-18. Yang et al. evaluated the importance of NLRP3 inflammasome on the development of AR in human and mouse model of AR, showing that the level of IL-1β and IL-18 in the nasal lavage fluid (NLF) was greater in the AR patients than in healthy controls. However, the level of IL-1β was lower in patients with mild persistent AR than in patients with moderate to severe persistent AR. Furthermore, in the inferior turbinate mucosal tissue, the level of IL-1β and Caspase-1, was higher in patients with AR in comparison to healthy control (30).

The OVA-induced AR mouse model successfully mimic AR as mice have characteristic symptoms of AR such as scratching and sneezing. It is also a good model to study the effect of NLRP3 inflammasome on AR pathogenesis. Yang et al. showed that AR mice had increased levels of IL-1β during AR development and enhanced levels of ASC, Caspase-1 and IL-1β in nasal epithelial tissue when compared with control mice (30). Together, the results from AR patients and the AR mouse model indicated the crucial role of NLRP3 inflammasome activation in AR development. However, the role of NLRP3 inflammasome in the pathogenesis of AR is still not fully explained. Subsequent results obtained by Yang et al. showed that the absence of NLRP3 (*Nlrp3^-/-^ mice)* affects the development of AR as the *Nlrp3^-/-^* mice had decreased AR symptoms of sneezing and scratching compared to wild-type mice (WT). The NLRP3 deficiency effectively inhibited the AR development in mice by reducing the Caspase-1 and IL-1β levels in the nasal mucosa. Moreover, its deficiency attenuated the production of IL-1β in NLF and the level of proinflammatory cytokines and chemokines i.e. IL-6, CXCL9, and CXCL10 in the nasal mucosa. Furthermore, the production of IL-4, IL-5, and IL-13 related to eosinophil activation was inhibited in the *Nlrp3^-/-^* mice. Since AR is an IgE-mediated type I allergic reaction, the level of IgE in the serum was measured, but no significant differences were observed between *Nlrp3^-/-^* and WT mice. However, it was found that the level of the gasdermin (GSDMD-N), the marker of cell pyroptosis, was significantly reduced in the nasal mucosa of *Nlrp3^-/-^* mice. Pyroptosis is a type of cell death caused by inflammasome activation, protecting against infectious agents and causing tissue damage. Pyroptosis is a possible trigger of mucosa injury with a simultaneous increase of IL-1β, Caspase-1, and gasdermin observed among AR patients and in mouse model of AR (30). Additionally, Yang et al. showed that the accumulation of ASC-specks and the associated pyroptosis of nasal epithelium contributed to the exacerbation of inflammation during AR development. They applied LPS and ATP on THP-1 cells prior to the isolation of the ASC-specks and next, the RPMI 2650 cell line was stimulated with ASC-specks. Results indicate that ASC-specks formed around the cell membrane of THP-1 cells after inflammasome activation and the RPMI 2650 epithelial cell line could uptake ASC-specks from the supernatant. Moreover, the production of IL-1β, GSDMD-N, Caspase-1 and the release of LDH (% of lactate dehydrogenase release serves as a marker of pyroptosis in cell lines) were increased in RPMI 2650 cell line after ASC-speck stimulation. This suggested that nasal epithelium pyroptosis could be induced by ASC-specks accumulation and this might enhance the inflammation (30). These data demonstrate that NLRP3 inflammasome activation contributes to AR development, however, independently of IgE production (30).

#### 3.1.3 The NLRP3 inflammasome antagonists

Yang et al. decided to investigate the specific Caspase-1 inhibitor Belnacasan as a potential treatment target for AR. The results showed that the administration of Belnacasan into mice with OVA-induced AR inhibited the AR symptoms i.e. sneezing and scratching compared with ATP administration which overactivated inflammasome and aggravated AR symptoms. Moreover, Belnacasan decreased the level of proinflammatory cytokines and chemokines i.e. IL-6, CXCL9, and IL-4 at mRNA levels, which indicates that inhibition of NLRP3 inflammasome activity may alleviate the symptoms of AR (30).

Likewise, Zhang et al. reported that administration of another selective NLRP3 inflammasome inhibitor (MCC950) to mice with OVA-induced AR, reduced the frequencies of AR symptoms (i.e. sneezing and nasal rubbing) in a dose-dependent manner in comparison with the WT group (31). The treatment with MCC950 caused a significant reduction in OVA-specific IgE levels in both, serum and NLF. It decreased the number of total eosinophils, macrophages, neutrophils and lymphocytes in NLF as well. Furthermore, the effect of MCC950 was investigated in nasal tissue. Eosinophils, goblet cells, NLRP3, Caspase-1, ASC, IL-1β, and IL-18 levels were significantly increased in nasal tissue of AR mice compared with control mice, but treatment with MCC950 significantly attenuated this effect. Moreover, the level of NLRP3^+^ cells was correlated with the presence of Caspase-1, ASC, IL-1β and IL-18 positive cells (31). Finally, the NLRP3, Caspase-1, ASC, IL-1β, and IL-18 were determined in spleen mononuclear cells and the supernatants after treatment with the inflammasome antagonist. It was reported that MCC950 significantly decreased the protein presence and mRNA expression for all determined molecules (31).

In conclusion, the data available indicate the crucial role of NLRP3 inflammasome in AR development. The usage of selective NLRP3 or Caspase-1 inhibitors, such as MCC950 or Belnacasan respectively, could be a promising strategy in AR treatment. However, further investigations confirming its effectiveness and safety are needed. A summary of the effects of inflammation’s inhibitors on allergic rhinitis is provided in **Table 1**.

**Table 1:** Inhibitors in respiratory diseases.

Disease	Research Model	Inhibitor	Treatment	Results	Ref.
AR	45 patients with AR 23 healthy control	Mito-TEMPO (mitochondrial ROS inhibitor)	Stimulation of PBMCs isolated from patients with Mito-TEMPO	Higher level of ROS in PBMCs from AR patients. Inhibition of IL-1β production in Mito-TEMPO treatment group.	(27)
AR	OVA-induced AR mouse model	Belnacasan (Caspase-1 inhibitor)	The intraperitoneal challenge with 5mg/ml Belnacasan solution for 7 days.	Inhibition of AR development and symptoms (sneeze and scratch). Downregulation of IL-6, IL-4, CXCL9 and Ear1 level.	(30)
AR	OVA-induced AR mouse model	MCC950 (NLRP3 inflammasome inhibitor) Dexamethasone (DEX)	Nasal administration of 200µg/400µg MCC950 for 8 days. Nasal administration of DEX (5mg/kg).	Decreased level of AR symptoms (sneeze, nasal rubbing). Inhibition of NLRP3, Caspase-1, ASC, IL-1β and IL-18 levels in nasal tissue and spleen mononuclear cells. Reduction in the levels of ECP, IL-4, IL-5, IL-13, IL-1β, and IL-18 in the nasal lavage fluid, and an increase in the level of IFN-γ.	(31)
AA	OVA-induced allergy mice model	Dexamethasone (DEX)	Intraperitoneal injection of DEX (2mg/kg) for 8 days.	Decreased the number of inflammatory cells infiltration and the levels of IL-1β, IL-18, IL-5, and IL-17 in BALF. Decreased the protein levels of NLRP3, Caspase-1, Procaspase-1, IL-1β, IL-6, IL-17, and TNF-α in DEX-treated mice lungs.	(32)
AA	House dust mite (HDM) model of mice allergic asthma	RRx-001 (anticancer agent)	Intraperitoneal injection of RRx-001 (10mg/kg) on days 7, 9, and 11 days.	Decreased the cell infiltration and goblet cells hyperplasia. Reduction the level of specific IgE in serum, and IL-4, IL-5, IL-6 and IL-13 levels in BALF and MNL cells.	(33)
AA	LPS/ATP or nigericin induced model of: J774A.1 (murine macrophages cell line) HMDMs (human monocyte-derived macrophages) Human blood neutrophils	OLT1177 (a β-sulfonyl nitrile compound)	Cell line stimulation with OLT1177.	Decreased the protein levels of Procaspase-1 and pro-IL-1β. Decreased the NLRP3 inflammasome formation. Decreased the protein levels of Caspase-1 and IL-1β.	(34)
AA	OVA-induced mice allergic asthma model House dust mite (HDM) induced mice allergic asthma model OVA-induced mice allergic asthma model with poly(I:C)-triggered exacerbation	OLT1177 (a β-sulfonyl nitrile compound)	Intraperitoneal treatment of OLT1177 (60mg/kg).	Decreasing the cytokine levels (IL-1β, IL-4, IL-5, IL-13, IL-6 and TNF-α) in BAL and expression level of *Nlrp3* and Caspase-1. Decreasing the number of inflammatory cells in BAL and lung tissue and airway hyper-responsiveness AHR.	(35)
AA	OVA-induced mice allergic airway inflammation model	ABA (abscisic acid)	Intraperitoneal injection of ABA (60mg/kg) for 7 days.	Decreased inflammatory cells infiltration, goblet cells hyperplasia in the lung and infiltration of eosinophils in BALF. Reduction of OVA-specific IgE in serum and cytokines levels (IL-4, IL-5, and IL-13). Reduction of the levels of NLRP3, Caspase-1 and IL-1β in lung tissue.	(36)
AA	OVA-induced mice allergic airway inflammation model	MCC950 (NLRP3 inflammasome inhibitor) Sevoflurane	Intraperitoneal injection MCC950 (10mg/kg) or 3% sevoflurane for 7 days.	Decrease level of inflammatory cells, and serum IgE level. Decreased cytokines IL-4, IL-13 levels in BALF and increased level of IFN-γ. Reduction in NLRP3 protein level.	(37)
COPD	16HBE (Human bronchial epithelial cell line)	VX-765 (a specific Caspase-1 inhibitor)	Cell line stimulation with CSE and CSE/VX-765.	Decrease level of Caspase-1, IL-1β, IL-18, and LDH after CSE/VX-765 treatment.	(38)
COPD	Cigarette smoke induced COPD mice model	LP17 - the specific TREM-1 inhibitor	Instillation intratracheally LP17 (1mg/kg) before CS exposure for 6 days a week for 15 weeks.	Decrease level of IL-1β, IL-18, TNF-α, NLRP3, ASC, Procaspase-1, Caspase-1 and GSDMD after LP17 treatment compared to COPD mice. Reduction of bronchial wall thickening and inflammatory cell infiltration after LP17 treatment compared to COPD mice.	(39)
COPD	Cigarette smoke induced COPD mice model	Melatonin	Intraperitoneal injection of melatonin (2.5 - 20mg/kg) before CS exposure, 6 day/week up to 4 weeks.	Decrease level of IL-1β, TNF-α in BALF, and protein level of NLRP3, Caspase-1 and IL-1β in mice lungs after melatonin treatment.	(40)
COPD	LPS/cigarette smoke rat model of COPD	Melatonin	Intraperitoneal injection of melatonin (10mg/kg/day) before CS exposure for 28 days.	Decrease level of IL-1β in BALF and, NLRP3, Caspase-1 and ASC in lungs after melatonin treatment.	(41)
COPD	LSP-induced COPD mice model	Histidine	Administration of histidine (2.0g/l) directly with the drinking water for 3 weeks.	Decrease levels of IL-1β, MCP-1, IL-6, and TNF-α in BALF, and levels of NLRP3, Procaspase-1, Caspase-1, and IL-1β in lung tissue.	(42)
COPD	LSP-induced COPD mice model	MCC950 Dexamethasone (DEX)	Intraperitoneal administration or nasal drip administration of MCC950 (10 or 50mg/kg) or DEX (0,5mg/kg).	Decrease the mRNA levels of *il-8, tgf-β1, il-1β* and the protein level of IL-18 and IL-1β in lung tissue after MCC950 or DEX treatment. Decrease the number of neutrophils, macrophages, and lymphocytes in BALF.	(43)
COPD	LPS/cigarette smoke mouse model of COPD	Lipoxin receptor agonist BML-111 Dexamethasone (DEX)	Intraperitoneal administration of BML-111 (1mg/kg and 10mg/kg) or DEX (2mg/kg) on the first day of the experiment.	Decrease level of IL-1β in BALF and protein level of NLRP3, Caspase-1 and IL-1β in lung tissue after BML-111 and DEX administration.	(44)
COPD	BEAS-2B (Human bronchial epithelial cell line)	(–)-Epicatechin – a type of flavonoid	Cell line stimulation with CSE and (–)-Epicatechin.	Decrease the protein level of NLRP3, Caspase-1, GSDMD, IL-18, and IL-1β and LDH levels after (–)-Epicatechin treatment.	(45)

### 3.2 NLRP3 inflammasome in allergic asthma (AA)

#### 3.2.1 Allergic asthma - A disease overview

Asthma and rhinitis are common comorbidities, however, studies have shown that both diseases differ in their origin (21, 46). According to The Global Initiative in Asthma (GINA), asthma is a long-lasting inflammatory state in a lower airway, which leads to narrowed bronchial tubes, thickening of the airway wall, increased mucus production, and hyperresponsiveness. The main symptoms include, for instance, wheezing, cough, short breath, and chest tightness, and their intensity varies (47). The increase of new cases of asthma is noted worldwide. It is predicted that by 2025 around 100 million people will suffer from asthma globally (48). Asthma can be triggered by many factors, such as allergens, pollutants, cigarette smoke, temperature variations, stress, physical exercise, various infections, and others (49). Mortality constantly rises and is higher in the women population (49).

Asthma, similar to rhinitis, is a heterogenous term, describing a condition with similar symptoms. However, the background, onset of the disease, its severity, treatment response, and related factors (obesity, cigarette smoke, physical exercise, aspirin intake) may vary. In clinical practice, determining the type of asthma allows for personalised medicine and better therapeutic outcomes (50–52). There are many asthma classification systems. The most common asthma classification is based on general background: allergic and non-allergic asthma, nevertheless, in clinical practice its diagnosis can be difficult (53). Allergic asthma is an atopic form, which usually has an early onset. It is accelerated after contact with an allergen, the sIgE and skin prick test are positive and its response to the steroid treatment is quite good. Non-allergic asthma is a non-atopic form with a late-onset and negative sIgE/skin prick test. It is characterised by a more severe course and poorer asthma-related quality of life (54, 55). Another classification is based on the onset of the disease. Adults with early-onset have an atopic background and more frequent symptoms. Adults with late-onset are non-atopic usually, with a worse course of the disease, women predominant, smoke-related (56). The cluster analysis of clinical features among large cohorts of asthmatics patients allows for determining more differential categories, including the severity of the disease and frequency of the symptoms, physical activity, obesity, and others (52, 57). However, a classification based on the immunological/cellular background seems to be especially interesting, because it clearly shows the complexity of asthma. It includes eosinophilic, neutrophilic, mixed, and paucigranulocytic subgroups of asthma (51). Recently, molecular classification, including endotype associated with Th2 and without Th2 response, has gained importance, mainly because it helps to determine the pathomechanism of the disease (50, 58–60). However, the above-mentioned varied clinical forms of asthma and the multitude of classification forms indicate how important it is to determine the pathomechanism and find a common denominator between different types of the disease.

#### 3.2.2 Allergic asthma - link with NLRP3 and IL-1β

Nowadays, much attention has been paid to the role of the inflammasome in asthma and its relationship with asthma phenotypes (61). The results obtained by Besnard et al. (62) showed that NLRP3 inflammasome is involved in the development of allergic airway inflammation. The authors based their research on the NLRP3 gene-deficient (*Nlrp3^-/-^)* mice in an OVA-induced allergic lung inflammation model in the absence of aluminium adjuvants. The increased infiltration of cells, mostly eosinophils in BAL (Bronchoalveolar lavage) and eosinophils and lymphocytes in lung tissue, in OVA-challenged WT mice was shown when compared with *Nlrp3^-/-^* mice. Moreover, the level of infiltrated eosinophils and lymphocytes was reduced in *Nlrp3^-/-^* mice compared with OVA-challenged WT mice (62). The increased level of cells in OVA-challenged WT mice was correlated with mucus hypersecretion, thickening of the epithelium, hyperplasia of goblet cells and smooth muscle, while in OVA-challenged *Nlrp3^-/-^* mice lung inflammation and mucus overproduction were decreased (62). Interestingly, the reduced level of IL-1β and IL-6 in *Nlrp3^-/-^* mice lung homogenate compared with OVA-stimulated WT mice, as well as decreased level of Th2 cytokines i.e. IL-5 in BAL, and TSLP, IL-33 in the lungs was observed. In the culture supernatants of mediastinal lymph nodes (mLN) from *Nlrp3^-/-^* mice, the levels of IL-4 and IL-13 after antigen restimulation also were lower when compared with WT mice. Serum level of OVA-specific IgE was significantly diminished in *Nlrp3^-/-^* mice than in WT mice, as well as the expression level of RANTES, eotaxin and TARC. Additionally, lack of NLRP3 affected CD4^+^ T cells recruitment and dendritic cells (DC) migration compared with WT mice (62).

The impact of NLRP3 inflammasome on the development of allergic airway inflammation was investigated by Guan et al. (32) in the OVA-induced allergy mouse model, with a particular interest in the effect of Dexamethasone (DEX) (mainstay treatment for asthma). In the DEX-treated mice group, the number of total leukocytes, eosinophils, monocytes, neutrophils and lymphocytes in BALF was significantly decreased compared with the OVA-induced mice group. Contrary to control mice, in DEX-treated mice, the infiltration of inflammatory cells and goblet cells counts were reduced, and the airway thickening effect was alleviated (32). Moreover, the level of inflammatory interleukins, those dependent on the NLRP3 inflammasome activity (IL-1β, IL-18), and other inflammatory factors (IL-5 and IL-17) was lowered in BALF (32). Additionally, the level of NLRP3, Caspase-1, Procaspase-1, IL-1β, IL-6, IL-17, and TNF-α in the lungs were also significantly downregulated by DEX treatment (32). The results obtained by Guan et al. (32) are in line with previous research carried out by Besnard et al. (62), which indicated that NLRP3 inflammasome activity had a significant influence on the development of allergic airway inflammation. Moreover, the results showed that treatment with DEX significantly reduced the symptoms of allergic airway inflammation and the activity of the NLRP3 inflammasome. It may indicate that DEX is acting by inhibiting inflammasome activity.

Furthermore, the role of NLRP3 inflammasome was confirmed in the development of allergic asthma induced by house dust mite (HDM) (33). Authors showed that the HDM challenge promoted the activation of NLRP3 inflammasome in the lungs of asthmatic mice and this process was dependent on alveolar macrophages. Mice with HDM-induced allergic asthma had an increased level of cells (eosinophils, alveolar macrophages and neutrophils) in BALF compared with control mice. In lung lysate, HDM stimulation caused a local secretion of inflammatory cytokine IL-1β. Moreover, from among five receptor proteins: NLRP1, NLRP3, NLRC4, AIM2, and pyrin, only the NLRP3 was significantly increased in the HDM-induced mouse model. In addition, the knockout of NLRP3 attenuated the level of IL-1β and Caspase-1 cleavage in the asthmatic lung compared with WT asthmatic mice (33) underlying the involvement of the NLRP3 inflammasome in the development of allergic diseases. The flow cytometric results showed that NLRP3 activity increased in alveolar macrophages, neutrophils, and dendritic cells among asthmatic mice. Additionally, Caspase-1 and IL-1β increased significantly in alveolar macrophages of asthmatic mice compared with a control group (33). The level of eosinophils and neutrophils in BALF, as well as the level of specific anty-HDM IgE in serum, were decreased in *Nlrp3^-/-^* mice after HDM exposure compared with the asthmatic WT group. This denotes attenuation in the Th2 response. Measurement of cytokines in BALF and restimulated mediastinal lymph nodes (MLNs) from HDM-treated *Nlrp3^-/-^* mice showed a significant decrease in IL-4, IL-5, IL-6, and IL-13 production compared with HDM-treated WT mice. However, the difference in the level of IFN-γ was not observed. Moreover, the mucus production and goblet cells hyperplasia induced by HDM treatment in lungs of *Nlrp3^-/-^* mice were diminished compared with WT mice. Additionally, it was confirmed in the *Nlrp3^Y30E/Y30E^* mutant mouse model that active NLRP3 inflammasome promoted inflammation and pathological tissue damage in asthma in an inflammasome-dependent manner. This mutation does not affect the NLRP3 expression but inhibits the activation of NLRP3 inflammasome through blockade dephosphorylation at Tyr30 of NLRP3 (33). Additionally, Ma et al. investigated the effect of RRx-001 (1-bromoacetyl-3,3-dinitroazetidine) – the well tolerated anticancer agent, on the development of HDM-induced asthma. The RRx-001 treatment significantly inhibited HDM-induced infiltration of eosinophils, neutrophils and lymphocytes, decreased the level of total and HDM-specific IgE in serum and attenuated the goblet-cells hyperplasia. Moreover, RRX-001 stimulation decreased the levels of IL-4, IL-5, IL-13, and IL-6 in BALF and MLN cells compared with WT asthmatic mice. These findings are consistent with results from *Nlrp3^-/-^* mice (33). The data available indicate the importance of NLRP3 in the allergic response and underlines a potential therapeutic effect of RRx-001 on asthma development.

#### 3.2.3 The NLRP3 inflammasome antagonists

There is ample evidence that the activation of NLRP3 inflammasome acts directly on the development of allergic asthma. Nowadays, novel specific NLRP3 inflammasome antagonists, which could reduce inflammation and support therapy in allergic diseases, have been sought. Marchetti et al. (34) showed that OLT1177 – a β-sulfonyl nitrile compound is a potentially good inhibitor of the NLRP3 inflammasome in allergic diseases. *In vitro*, the NLRP3 inflammasome was activated by LPS/ATP or LPS/nigericin stimulation in the murine macrophages cell line J774A.1 and human monocyte-derived macrophages HMDMs. It was shown that OLT1177 reduced the formation of the NLRP3 inflammasome in a highly specific manner and did not affect NLRC4 and AIM2 inflammasomes. The usage of OLT1177 decreased the level of secreted IL-1β, active Caspase-1 in the cells’ lysate, and the level of released LDH. However, the intracellular level of Procaspase-1 and pro-IL-1β as well as mRNA of *nlrp3*, *asc*, *caspase-1*, *il-1β* and *il-18* remained unchanged. These results suggest that OLT1177 could reduce inflammation by selective decreasing NLRP3 inflammasome formation and reducing the secretion of IL-1β (34). Subsequently, the inhibitory effect of OLT1177 was confirmed *ex vivo*, in freshly obtained human blood neutrophils followed by LPS/ATP stimulation. OLT1177 inhibited the IL-1β secretion and Caspase-1 activity with no effect on *il-1β*, *caspase-1* and *nlrp3* mRNA levels. Furthermore, the research confirmed the positive effect of OLT1177 on reducing the IL-1β level and its safety in use among humans affected by the cryopyrin-associated periodic syndrome (CAPS) (34).

In an OVA-induced model of asthma (35), treatment with OLT1177 reduced the secretion of IL-1β, Th2 type cytokines (IL-4, IL-5, IL-13) and proinflammatory cytokines (IL-6, TNF-α) in BAL. Simultaneously, the level of *Nlrp3* expression and Caspase-1 activation in the lungs were diminished and the level of eosinophils in BALF was reduced. In airway tissue, a decrease in the infiltration of immunological cells and attenuation of goblet cells’ hyperplasia were observed. Moreover, the symptoms of hyperresponsiveness (AHR) were attenuated. Further, in HDM-induced allergic asthma, the intraperitoneal administration of OLT1177 resulted in a decrease in the number of eosinophils and neutrophils in BAL and the number of inflammatory cells in the lungs tissue. Consistent results were obtained for the OVA-induced allergic asthma model with poly(I:C)-triggered exacerbation (35). The above results enforce that OLT1177 efficacy does not depend on the route of administration or the model chosen.

Besides the synthetic antagonists, the naturally originated sources of inflammasome inhibitors seem to be very promising as well. Recently, abscisic acid (ABA) has started to attract more attention due to its anti-inflammatory and wound healing capacity (63, 64). ABA is a plant phytohormone that regulates the plant’s response to environmental stress and regulates seed dormancy and germination. However, ABA is synthesised endogenously in animal and human cells such as granulocytes and macrophages. Zhao et al. (36) investigated the effect of ABA on NLRP3 inflammasome activity in the OVA-induced allergic airway inflammation mouse model (36). The results underlined its inhibitory effect on the NLRP3 inflammasome activation, which was reflected in the reduction of NLRP3, Caspase-1 and IL-1β levels in both models. In an *in vivo* experiment, the administration of ABA significantly decreased the infiltration of inflammatory cells and hyperplasia of goblet cells in the lungs, as well as reduced the number of eosinophils in BALF. ABA treatment alleviated OVA-specific IgE in serum and Th2 cytokines levels such as IL-4, IL-5, and IL-13 (36). The anti-inflammatory effect of ABA was mediated by PPAR-γ activity. PPAR-γ is a nuclear receptor that promotes the anti-inflammatory activation of M2 macrophages in mice. It was shown that the expression of PPAR-γ increased in OVA-included allergy mice after treatment with ABA compared with OVA-induced allergy mice without treatment. Strikingly, the additional application of GW9662, an antagonist of PPAR-γ, abrogated the ABA inhibitory effect on mucus production and infiltration of inflammatory cells. Moreover, GW9662 reversed the inhibition of IL-5, IL-4 and IL-13 cytokines. Simultaneously, administration of GW9662 and ABA led to a complete loss of the inhibitory effect of ABA on the NLRP3 inflammasome activity increasing NLRP3, Caspase-1 and IL-1β levels (36).

There is more study which presents the capacity of various factors for the NLRP3 inflammasome inhibition, i.e. decreasing the apolipoprotein E gene expression (65), or the melatonin participation in the TLR2 receptor inhibition loop involved in the NLRP3 activation pathway of the inflammasome (14). Also, sevoflurane, a commonly used volatile anaesthetic, has a comparable to MCC950 favourable effect on NLRP3 inflammasome inhibition and decreases asthma symptoms (66). A summary of the effects of various inhibitors on allergic asthma is presented in **Table 1**. The above-presented examples provide evidence of a significant influence of the NLRP3 inflammasome activity on the development of allergic asthma and mouse allergic airway inflammation. These findings open up new therapeutic possibilities in allergic-based diseases. Nevertheless, further research is required to decipher the exact role of NLRP3 inflammasome in the pathomechanism of allergic asthma.

### 3.3 NLRP3 inflammasome in Chronic Obstructive Pulmonary Disease (COPD)

#### 3.3.1 Chronic Obstructive Pulmonary Disease - A disease overview

According to the Global Initiative for Chronic Obstructive Lung Disease (GOLD) (67), COPD is a preventable and treatable disease, which concerns around 210 million people worldwide (68). It has a complex background, which includes the interaction of the external and internal factors i.e. genetic susceptibility, anatomical abnormalities in an airway tract and accompanying diseases such as asthma or allergy (69–73). The exact pathomechanism is still not fully understood. It is assumed that long-term exposure to cigarette smoke, as well as biofuel particles and air pollution, lead to inflammation, fibrosis, airway/alveolar abnormalities, and airflow limitation among predisposed patients (67). The main symptoms include dyspnoea, cough, sputum production, as well as a tight feeling in the chest. The heterogeneity of COPD and the lack of unambiguous diagnostic markers make the differential diagnosis necessary. The determination of COPD’s phenotypes allows for a better understanding of the disease, more precise diagnosis, and pharmacological management. The basic and widely accepted phenotypes include chronic bronchitis, emphysematous, rare or frequent exacerbator and asthma-COPD-overlap. However, a clinical picture of the disease can be more complex, therefore additional (emerging) phenotypes have been introduced e.g. pulmonary cachexia, overlap COPD and bronchiectasis, α1-antitrypsin deficiency, no smoking COPD and others (74). Similarly to AR and asthma, COPD is characterised by complex and not fully clear pathomechanism, a varied clinical picture and a lack of targeted therapy. This diversity has resulted in an increasing interest in research into strategic inflammatory components, including inflammasome and IL-1β.

#### 3.3.2 Chronic Obstructive Pulmonary Disease - Link with NLRP3 and IL-1β

Since COPD has an inflammatory background, Yu et al. (75) determined the cytokines profile among patients hospitalised due to acute exacerbation of COPD. The cytokine range included Th1 (TNF-α, IFN-γ, IL-2), Th2 (IL-4, IL-5, IL-17E), Th17 (IL-6, IL-17A, IL-21, IL-22, IL-23) and Treg (IL-10) related factors. Although, the level of the IL-17E seemed to be prominent, the cluster analysis of the study group (n=82) allowed to set down 3 subtypes: Th1 high-Th17 high (cluster 1), Th1 low- Th17 low (cluster 2), and Th1 high-Th17 low (cluster 3), which correlated with different clinical pictures. The decrease in sputum of IL-17A concentration was associated with the increase in the frequency of exacerbation episodes. The group of patients with the profile Th1 high-Th17 low indicated a worse course of the disease, expressed by longer non-invasive artificial ventilation, hospital stay and more frequent exacerbation episodes (75). Contrary, Sun et al. (76) reported that the profile of Th response changes during the disease course. The acute stage of COPD is characterised by the Th2 response, while in the remission phase the Th1 response is predominant, which could arise from the treatment (76). Recently, the differential gene expression analysis has been performed among the group of patients with (n=61) and without (n=227) COPD (77). The authors reported different patterns in gene expression (up-regulation of 38 genes and down-regulation of 114 genes COPD group compared with the healthy control group) and strong inflammatory background of the disease. According to the authors, IL-1β seems to be especially important. It was indicated that it is up-regulated only in the small airway epithelial cells and it correlates positively with COPD status as well as its clinical biomarkers. The authors proposed that IL-1β can be a novel player in airway inflammation in COPD (77). Indeed, Wang et al. (37) confirmed this suggestion based on the results obtained from examining smokers with normal pulmonary function and patients in various stages of COPD - acute exacerbation, recovery and stable stage. The total number of cells and the number of neutrophils, lymphocytes and macrophages in BALF were significantly increased in patients with acute exacerbation stage compared with smokers. Among patients in the recovery stage and the stable stage, the level of cells was significantly reduced compared with the patients in the acute exacerbation stage but still higher than in the smokers. The mRNA measurement of *nlrp3*, *asc*, *caspase-1*, *il-1β* and *il-18* in the PBMC and bronchial tissues of COPD patients revealed a significant increase in all groups of COPD patients compared with the smoker group. In contrast, the mRNA level of *nlrp3*, *caspase-1*, *asc*, *il-18* and *il-1β* in recovery and stable COPD patients were decreased compared with acute exacerbation COPD patients. The levels of IL-18 and IL-1β in BALF and serum were significantly elevated in all groups of COPD patients compared with smokers, although the levels differed significantly between the groups. These results showed that local and systemic airway activation of the NLRP3 inflammasome could lead to the exacerbation of COPD (37). Also, the *in vitro* models of COPD suggested that NLRP3 inflammasome may participate in the development of COPD. Results of A549 cell line stimulation (pulmonary adenocarcinoma derived cell line) with cigarette smoke extract (CSE) showed an increase in the level of NLRP3 protein and IL-1β production in a dose-dependent manner (78).

Extensive research on the function of the NLRP3 inflammasome in COPD development was conducted on a human bronchial epithelial 16HBE cell line (38). In the study, a 16HBE cell line was stimulated with CSE, which imposes a toxic effect. It was reported that CSE significantly increased the level of the cleaved form of Caspase-1, GSDMD, IL-1β, and IL-18 compared with the control group. In addition, the level of LDH increased as a result of pyroptosis and was positively correlated with CSE dose (38). To determine if pyroptosis is Caspase-1 dependent, VX-765 (a specific Caspase-1 inhibitor), was used in the experiment. VX-765 co-treatment with CSE decreased the levels of Caspase-1, IL-1β, IL-18, and LDH compared with the CSE treatment group. Additionally, the protein and mRNA levels of *caspase-1* and *Nlrp3* were significantly elevated after CSE stimulation. Transfection with siNLRP3 abolished these effects and declined the NLRP3 and Caspase-1, both on mRNA and proteins level. Moreover, the mRNA levels of *il-1β* and *il-18* were reduced by siNLRP3 (38). These findings suggest that CSE stimulation activates the NLRP3 inflammasome and induces pyroptosis in a Caspase-1-dependent manner, which has a significant impact on the development of COPD.

#### 3.3.3 The NLRP3 inflammasome antagonists

Wang et al. (39) also investigated the NLRP3 inflammasome mediated pyroptosis and its impact on COPD development using LP17 - the specific TREM-1 inhibitor. They noticed that LP17 application successfully reversed the COPD symptoms in COPD mice, through bronchial wall thickening and attenuation of inflammatory cells infiltration. A significant reduction in the number of macrophages in the lungs among COPD mice was observed. Additionally, Wang et al. noticed an increased level of IL-1β, IL-18, and TNF-α in the lung tissue and BALF of COPD mice compared with control mice. Moreover, the levels of NLRP3 inflammasome-related proteins: NLRP3, ASC, Procaspase-1, Caspase-1, and GSDMD were significantly increased in lung tissue of COPD mice, whereas LP17 administration abolished these effects and decreased the level of these proteins. In summary, these results indicate that TREM-1 inhibition attenuated NLRP3 inflammasome activity and inflammasome-dependent pyroptosis (39). Interestingly, the impact of melatonin on the COPD and NLRP3 inflammasome was investigated as well (40, 41). Mahalanobish et al. (40) showed that exposure to CSE increased the total cell count in BALF, neutrophils’ infiltration in the lungs and the LDH and myeloperoxidase (MPO) secretion in the mouse model of COPD. Moreover, after CSE exposure, the levels of TNF-α and IL-1β in BALF elevated. Also, the protein levels of NLRP3, Caspase-1 and IL-1β were enhanced in the mice lungs after CSE exposure. However, the melatonin administration successfully reversed the effects induced by CSE and reduced the level of inflammatory molecules in the CSE-exposed group (40). Since endoplasmic reticulum (ER) stress and oxidative stress also influence inflammasome activation, the effect of melatonin on these factors was investigated. The results showed that melatonin decreased levels of ER stress markers and mitochondrial damage markers, which were upregulated in CSE-treatment mice. These results indicate that NLRP3 inflammasome is a key player in COPD development, and melatonin has a great potential to inhibit COPD symptoms (40). Similarly, Peng et al. (41) showed that CSE exposure led to an increase in the number of total inflammatory cells and the percentage of neutrophils in BALF compared with the control group in a rat model of COPD. The level of IL-1β was also significantly increased. Additionally, the levels of NLRP3, cleaved Caspase-1 and ASC in lung tissue were significantly higher compared with control. This is in line with the results of Mahalanobish et al. (40) which indicated that the treatment with melatonin significantly decreased these effects and reduced the levels of IL-1β, NLRP3, cleaved Caspase-1 and ASC (40). Therefore, the effectiveness of melatonin in inhibiting the activity of the NLRP3 inflammasome and reducing the symptoms of COPD was confirmed. In addition, Peng et al. (41) conducted experiments using EX527 (SIRT1 inhibitor, stress resistance and inflammation regulator) in a rat model of COPD showing the inhibitory effect of melatonin on the NLRP3 inflammasome activity depends on the SIRT1 pathway. Moreover, treatment with EX527 inhibited the effects of melatonin, and re-increased levels of NLRP3, Caspase-1, ASC and IL-1β (41).

Likewise, the inhibitory effect of histidine on LPS-induced COPD mice was investigated (42). Tian et al. (42) showed that LPS stimulation increased inflammatory cells’ infiltration into mice lungs, which was significantly attenuated by histidine treatment. Also, the histidine supplementation suppressed total cells and macrophages counts in BALF compared with LPS-induced mice. Additionally, the levels of chemokine MCP-1, as well as pro-inflammatory cytokines IL-6, TNF-α, and IL-1β, were increased in LPS-treatment mice, and histidine significantly ameliorated these effects. Furthermore, histidine treatment decreased levels of the NLRP3, Procaspase-1, Caspase-1, and IL-1β in lung tissue when compared to the LPS-induced group. The effect of SIRT1 expression was investigated as well. Similarly to previous findings, Tian et al. (42) showed, that in the LPS-induced mice COPD model, the SIRT1 expression was lower compared with the control group and the histidine supplementation significantly increased the SIRT1 level. Furthermore, the EX527 (SIRT1-specific inhibitor) treatment inhibited the histidine effect, decreased the level of SIRT1 and increased the NLRP3 level (42). This indicates that histidine inhibits the NLRP3 inflammasome activation *via* the SIRT1-dependent pathway.

Some investigations were also performed in mouse model of LPS-induced respiratory inflammation, which is a relevant component of COPD (43). In this model, a significant increase in the number of cells i.e. neutrophils, macrophages and lymphocytes in BALF was observed as well. Moreover, mRNA levels of *il-8*, *tgf-β1*, *il-1β* and the protein level of IL-18, and IL-1β in lung tissue increased significantly. In addition, serum levels of IL-1β and IL-18 were also increased, demonstrating the involvement of the NLRP3 inflammasome in the development of COPD (43). Interestingly, treatment with MCC950 (50mg/kg) or dexamethasone (DEX, 0,5mg/kg) as the nasal drip or intraperitoneal injection in mice with LPS-induced airway inflammation, significantly decreased the number of neutrophils, macrophages, and lymphocytes in BALF as well as mRNA levels of *il-1β*, *il-8*, and *tgf-β1*. Additionally, the protein levels of IL-18 and IL-1β in lung tissue as well as in serum were reduced significantly by MCC950 and DEX, indicating their potential to reduce the disease symptoms (43).

There are still many other studies showing the influence of various factors on the inhibition of the NLRP3 inflammasome in COPD, i.e. lipoxin receptor agonist BML-111 (44) or (–)-Epicatechin – a type of flavonoid (45). The above findings provide evidence of a significant influence of the NLRP3 inflammasome activity on the development of COPD and pointed out that inhibition of NLRP3 inflammasome activity efficiently ameliorates the symptoms of COPD (37–39, 41–43). A summary of the effects of various inhibitors on COPD is provided in **Table 1**.

## 4 A future perspective: Impact of probiotic bacteria on the NLRP3 inflammasome in the context of respiratory diseases

**Figure 3:**
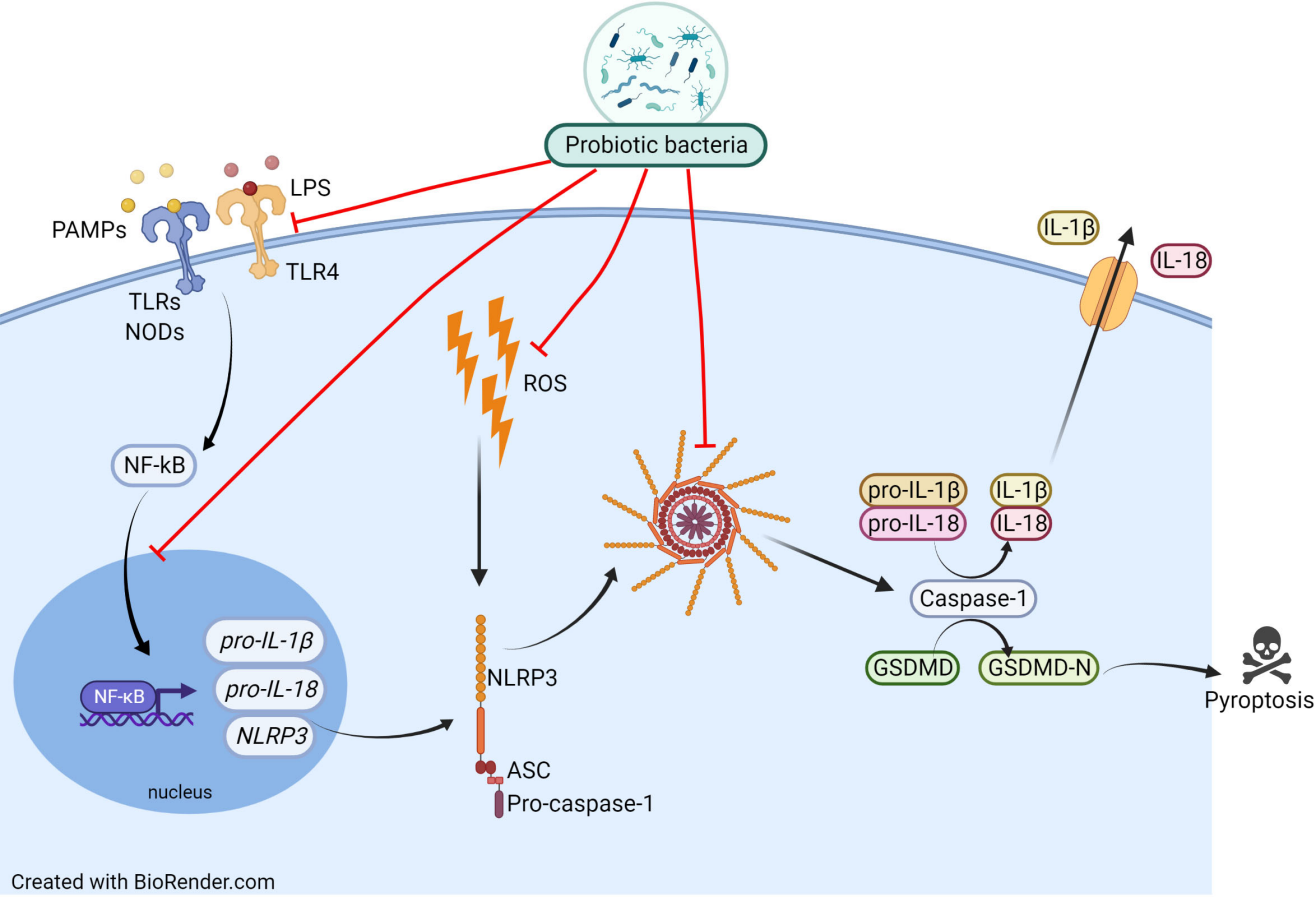
Proposed mechanism of probiotic actions on the NLRP3 inflammasome activation pathway. Inhibition of the NLRP3 inflammasome by probiotic bacteria could occur by inhibiting TLRs and NODs receptors, lowering their expression, downregulating NF-κB expression or inhibiting its movement to the nucleus. Probiotics may work by inhibiting inflammasome formation and Caspase-1 activation, which leads to a decrease in the level of IL-1β and IL-18 release. Potential inhibition of ROS production by probiotic bacteria would lead to a reduction in NLRP3 inflammasome activation.

Since NLRP3 inflammasome belongs to the PRRs family (recognizing, among others, the bacterial molecular patterns), especially interesting seems to be the probiotics’ impact on inflammasome activity. Recently, probiotics have become an important direction of research and seem to be promising in the support of respiratory disorders treatment. However, it has to be underlined that despite an enormous amount of research on the usefulness of probiotics in the treatment of respiratory diseases, no studies link the bacterial health-promoting effect to the inhibition of the NLRP3 inflammasome. The probiotics’ beneficial effect on allergic asthma and rhinitis has recently been well summarised by Jakubczyk et al. (79). The widely accepted probiotics’ mechanism of action in allergic disease is based on the bacterial ability to impact the host’s Th1/Th2 balance, induction of tolerance, changes in cytokines level (e.g IL-4), and in respective ratios IL-10/IFN-γ, Treg/TGF-β, as well as reduction of serum eosinophil level (80). It is highly probable that an administration of probiotic bacteria affects the inflammasome activity resulting in the reduction of airway inflammation and alleviation of the allergic disease’s symptoms. In **Figure 3** we presumed how probiotics may inhibit the NLRP3 inflammasome in the context of allergic diseases. Inhibition of the NLRP3 inflammasome by probiotic bacteria could occur through interaction *via* TLRs and NODs receptors, downregulation of NF-κB expression or inhibition of its movement to the nucleus. Probiotics may work by inhibiting inflammasome formation and Caspase-1 activation, which leads to a decrease in the level of IL-1β and IL-18 release. Potential inhibition of ROS production by probiotic bacteria would lead to a reduction in NLRP3 inflammasome activation.

Moreover, except allergic asthma and rhinitis, some of the bacterial strains have exhibited the ability to alleviate COPD symptoms as well (81). For example, stimulation of *Lactobacillus rhamnosus* NutRes1 and *Bifidobacterium breve* M-16V significantly declined the levels of IL-1β, IL-6, TNF-α, IL-10, and IL-23 in CSE-activated THP-1 cell line (82), whereas administration of *Lactobacillus rhamnosus* in CSE-induced COPD mice markedly decreased the pro-inflammatory cytokines: IL-1β, IL-6, TNF-α, IL-17, TGF-β in BALF, mRNA level of *nf-κB* and increased the level of IL-10 (83). Once again, similarly to allergic asthma and rhinitis studies, the direct probiotics’ impact on the NLRP3 inflammasome was not determined.

However, it is worth emphasising that the impact of the different probiotic strains on NLRP3 inflammasome has been confirmed in other diseases. Avolio et al. (84) indicated the beneficial effect of a probiotic mixture composed of *Streptococcus thermophilus*, *Lactobacillus bulgaricus*, *Lactococcus lactis* subsp. *lactis*, *Lactobacillus acidophilus*, *Streptococcus thermophiles*, *Lactobacillus plantarum*, *Bifidobacterium lactis* and *Lactobacillus reuteri* on the Syrian golden hamster with a high-fat diet (HFD) and unpredictable chronic mild stress (UCMS), in which the level of the IL-1β, Caspase-1, and NLRP3 were decreased in hypothalamus and blood of both HFD and UCMS hamsters after treatment with these bacteria (84). Next, it was also shown that the treatment of HFD mice with bacteriocin PJ4 from *Lactobacillus helveticus* significantly decreased the expression level of *Nlrp3* and secretion level of IL-1β, IL-6, TNF-α, IFN-γ, and MPC-1 (85). Additionally, in DSS-induced acute colitis in mice, pre-treatment with *E. faecalis* affected NLRP3 inflammasome activation and decreased the level of cleaved Caspase-1 and IL-1β in colon tissue resulting in an improvement of the disease score (86). In the model of *Salmonella infantis*-induced diarrhoea, the mRNA level of *asc*, *caspase-1*, *il-18*, and protein levels of NLRP3, ASC, and Caspase-1 were lowered after *Lactobacillus johnsonii* L531 administration (87). Furthermore, Li et al. in the bovine mastitis model induced by *E*. *coli* indicated that the high level of NLRP3, ASC, Caspase-1 and the apoptosis proteins i.e. BAX and Caspase-3 were diminished by pre-treatment with *L. rhamnosus* GR-1. Interestingly, *L. rhamnosus* GR-1 alleviated the ROS production and H_2_O_2_-induced NLRP3 inflammasome activation (88).

This indicates that studying the effects of probiotics on the inflammasome is not a new field. However, in respiratory diseases it is still a completely unknown niche. Further research devoted to the probiotic-inflammasome interaction could help to define the mechanism of action triggered by beneficial bacteria. In turn, this will allow developing the new therapeutic strategies for respiratory disorders.

## 5 Conclusion

To summarise, the activity of the NLRP3 inflammasome seems to play a crucial role in the development of inflammation-based respiratory disorders such as allergic rhinitis allergic asthma, and COPD, and significantly exacerbates the development of these diseases. Despite the differences in pathomechanisms of discussed diseases, the NLRP3 inflammasome induces similar corollaries. Inflammation related to airway diseases depend on many internal and external factors including immunological, macrobiotic, genetic, and environmental stimuli. Even though the mechanism of activation and action of the NLRP3 inflammasome is well understood, its influence on the development of respiratory diseases is not fully investigated. As the studies presented above show, there are many substances with NLRP3 inhibitory potential, which impose a beneficial effect on the disease symptoms attenuation. It is necessary to understand the exact path of this impingement, therefore, further research is essential. Especially interesting is the beneficial effect of probiotic bacteria on airway diseases. Numerous studies show a significant influence of probiotic supplementation on the symptoms alleviation of respiratory diseases, such as asthma, rhinitis, and COPD. However, there is still a lack of studies showing the influence of probiotic bacteria on the NLRP3 inflammasome activity in those disorders. It seems to be crucial to understand the influence of both probiotic bacteria and the NLRP3 inflammasome on the inflammatory airway diseases course. This opens a new research area for the treatment of respiratory disorders with an inflammatory background.

## Author contributions

Conceptualization, KL, DJ, and SG; Supervision, SG; Writing—original draft, KL and DJ; Review & editing, KL, DJ, and SG. All authors have read and agreed to the published version of the manuscript.

## Funding

This work was supported by grant co-founded by the National Science Centre of Poland under grant decision number UMO-2017/26/E/NZ7/01202.

## Acknowledgments

Figures were created using BioRender online software (https://biorender.com/).

## Conflict of interest

The authors declare that the research was conducted in the absence of any commercial or financial relationships that could be construed as a potential conflict of interest.

## Publisher’s note

All claims expressed in this article are solely those of the authors and do not necessarily represent those of their affiliated organizations, or those of the publisher, the editors and the reviewers. Any product that may be evaluated in this article, or claim that may be made by its manufacturer, is not guaranteed or endorsed by the publisher.

## Abbreviations

AA, Allergic Asthma; ABA, Abscisic acid; AHR, Hyperresponsiveness; AIM2, Absent-in-melanoma 2; APC, Antigen-presenting cell; AR, Allergic rhinitis; ARIA, The Allergic Rhinitis and its Impact on Asthma; ASC, Apoptosis-associated speck-like protein containing a caspase-recruitment domain; ATP, Adenosine triphosphate; BAL, Bronchoalveolar lavage; BALF, Bronchoalveolar lavage fluid; BAX, Bcl-2-associated X protein; BC, Black carbon; BML-111, 5(S),6(R),7-Trihydroxyheptanoic acid methyl ester; a lipoxin receptor agonist; BRCC, BRCA1-BRCA2-containing complex subunit 3; cAMP, Cyclic AMP; CARD, Cysteinyl aspartate-specific proteinase (caspase) recruitment domain; COPD, Chronic obstructive pulmonary disease; CSE, Cigarette smoke extract; CXCL, Chemokine (C-X-C motif) ligand; DA, Neurotransmitter dopamine; DAMPs, Damage-associated molecular patterns; DC, Dendritic cells; DEX, Dexamethasone; DUB, Deubiquitinating enzyme; ER, Endoplasmic reticulum; EX527, 6-Chloro-2,3,4,9-tetrahydro-1H-Carbazole-1-carboxamide; SIRT1 inhibitor, stress resistance and inflammation regulator; FBXO3, F-box O3; GINA, The Global Initiative in Asthma; GSDMD, Gasdermin D; GW9662, 2-Chloro-5-nitro-N-phenylbenzamide; HDM, House dust mite; HFD, High-fat diet; hNEC, Human nasal mucosa samples; IAR, Intermittent allergic rhinitis; IFI16, Interferon-inducible protein 16; IFN-γ, Interferon gamma; Ig, Immunoglobulin; IL, Interleukin; ILC2s, Type 2 innate lymphoid cells; JNK-1, c-Jun N-terminal kinase 1; LAR, Local allergic rhinitis; LDH, Lactate dehydrogenase; LP17, TREM-1 inhibitory peptide; LPS, Lipopolysaccharide; LRRs, Leucine-rich repeat domain; MCC950, 1-(1,2,3,5,6,7-Hexahydro-s-indacen-4-yl)-3-[4-(2-hydroxypropan-2-yl)furan-2-yl]sulfonylurea; MCP-1, Monocyte chemoattractant protein 1; mLN, Mediastinal lymph nodes; MLN, Mediastinal lymph node; MPO, Myeloperoxidase; MPC-1, Mitochondrial pyruvate carrier 1; mRNA, Messenger RNA; NAC, Nasal allergen challenge; NAC, N-acetyl-L-cysteine; ROS inhibitor; NACHT, Nucleotide-binding oligomerization domain; NAR, Non-infectious rhinitis; NLF, Nasal lavage fluid; NLR, NOD-like receptor; NLRC4, NLR family CARD domain-containing protein 4; NLRP1, NLR family pyrin domain containing 1; NLRP3, NLR family pyrin domain containing 3; NLRP6, NLR family pyrin domain containing 6; NF-κB, Nuclear factor kappa-light-chain-enhancer of activated B cells; NOD, Nucleotide oligomerization domain; OLT1177, Dapansutrile, a β-sulfonyl nitrile compound; OVA, Ovalbumin; PAMP, Pathogen-associated molecular pattern; PBMC, Peripheral blood mononuclear cell; PPAR-γ, Peroxisome proliferator activated receptor; PRR, Pattern recognition receptor; PYD, Pyrin domain; RANTES, Regulated upon Activation, Normal T cell Expressed, and Secreted; RNA, Ribonucleic acid; ROS, Reactive oxygen species; RRx-001, 1-bromoacetyl-3,3-dinitroazetidine; SIRT1, Silent information regulator 1; TARC, Thymus and activation-regulated chemokine; TGF-β1, Transforming growth factor beta; TLR, Toll-like receptors; TNF-α, Tumour necrosis factor α; TREM1, Triggering receptor expressed on myeloid cells 1; TRIM31, Tripartite Motif Containing 31; TSLP, Thymic stromal lymphopoietin; Tyr, Tyrosine; UCMS, Unpredictable chronic mild stress; WT, Wild type; YVAD, (2*S*)-2-[[(2*S*)-2-[[(2*S*)-2-[[(2*S*)-2-amino-3-(4-hydroxyphenyl)propanoyl]amino]-3-methylbutanoyl]amino]propanoyl]amino]butanedioic acid; VX-765, Belnacasan.

## Glossary

**Table:**

AA	Allergic Asthma
ABA	Abscisic acid
AHR	Hyperresponsiveness
AIM2	Absent-in-melanoma 2
APC	Antigen-presenting cell
AR	Allergic rhinitis
ARIA	The Allergic Rhinitis and its Impact on Asthma
ASC	Apoptosis-associated speck-like protein containing a caspase-recruitment domain
ATP	Adenosine triphosphate
BAL	Bronchoalveolar lavage
BALF	Bronchoalveolar lavage fluid
BAX	Bcl-2-associated X protein
BC	Black carbon
BML-111	5(S),6(R), 7-Trihydroxyheptanoic acid methyl ester a lipoxin receptor agonist
BRCC	BRCA1-BRCA2-containing complex subunit 3
cAMP	Cyclic AMP
CARD	Cysteinyl aspartate-specific proteinase (caspase) recruitment domain
COPD	Chronic obstructive pulmonary disease
CSE	Cigarette smoke extract
CXCL	Chemokine (C-X-C motif) ligand
DA	Neurotransmitter dopamine
DAMPs	Damage-associated molecular patterns
DC	Dendritic cells
DEX	Dexamethasone
DUB	Deubiquitinating enzyme
ER	Endoplasmic reticulum
EX527	6-Chloro-2,3,4 9-tetrahydro-1H-Carbazole-1-carboxamide
SIRT1 inhibitor	stress resistance and inflammation regulator
FBXO3	F-box O3
GINA	The Global Initiative in Asthma
GSDMD	Gasdermin D
GW9662	2-Chloro-5-nitro-N-phenylbenzamide
HDM	House dust mite
HFD	High-fat diet
hNEC	Human nasal mucosa samples
IAR	Intermittent allergic rhinitis
IFI16	Interferon-inducible protein 16
IFN-γ	Interferon gamma
Ig	Immunoglobulin
IL	Interleukin
ILC2s	Type 2 innate lymphoid cells
JNK-1	c-Jun N-terminal kinase 1
LAR	Local allergic rhinitis
LDH	Lactate dehydrogenase
LP17	TREM-1 inhibitory peptide
LPS	Lipopolysaccharide
LRRs	Leucine-rich repeat domain
MCC950	1-(1,2,3,5,6,7-Hexahydro-s-indacen-4-yl)-3-[4-(2-hydroxypropan-2-yl)furan-2-yl]sulfonylurea
MCP-1	Monocyte chemoattractant protein 1
mLN	Mediastinal lymph nodes
MLN	Mediastinal lymph node
MPO	Myeloperoxidase
MPC-1	Mitochondrial pyruvate carrier 1
mRNA	Messenger RNA
NAC	Nasal allergen challenge
NAC	N-acetyl-L-cysteine, ROS inhibitor
NACHT	Nucleotide-binding oligomerization domain
NAR	Non-infectious rhinitis
NLF	Nasal lavage fluid
NLR	NOD-like receptor
NLRC4	NLR family CARD domain-containing protein 4
NLRP1	NLR family pyrin domain containing 1
NLRP3	NLR family pyrin domain containing 3
NLRP6	NLR family pyrin domain containing 6
NF-kB	Nuclear factor k-light-chain-enhancer of activated B cells
NOD	Nucleotide oligomerization domain
OLT1177	Dapansutrile, a β-sulfonyl nitrile compound
OVA	Ovalbumin
PAMP	Pathogen-associated molecular pattern
PBMC	Peripheral blood mononuclear cell
PPAR-γ	Peroxisome proliferator activated receptor
PRR	Pattern recognition receptor
PYD	Pyrin domain
RANTES	Regulated upon Activation
Normal T cell Expressed	and Secreted
RNA	Ribonucleic acid
ROS	Reactive oxygen species
RRx-001	1-bromoacetyl-3, 3-dinitroazetidine
SIRT1	Silent information regulator 1
TARC	Thymus and activation-regulated chemokine
TGF-β1	Transforming growth factor β
TLR	Toll-like receptors
TNF-α	Tumour necrosis factor α
TREM1	Triggering receptor expressed on myeloid cells 1
TRIM31	Tripartite Motif Containing 31
TSLP	Thymic stromal lymphopoietin
Tyr	Tyrosine
UCMS	Unpredictable chronic mild stress
WT	Wild type
YVAD	(2*S*)-2-[[(2 *S*)-2-[[(2 *S*)-2-[[(2 *S*)-2-amino-3-(4-hydroxyphenyl)propanoyl]amino]-3-methylbutanoyl]amino]propanoyl]amino]butanedioic acid
VX-765	Belnacasan.
